# Analysis of Glioblastoma Patients' Plasma Revealed the Presence of MicroRNAs with a Prognostic Impact on Survival and Those of Viral Origin

**DOI:** 10.1371/journal.pone.0125791

**Published:** 2015-05-07

**Authors:** Ana Herman, Kristina Gruden, Andrej Blejec, Vid Podpečan, Helena Motaln, Primož Rožman, Matjaž Hren, Klemen Zupančič, Matija Veber, Urška Verbovšek, Tamara Lah Turnšek, Andrej Porčnik, Marjan Koršič, Miomir Knežević, Matjaž Jeras

**Affiliations:** 1 Blood Transfusion Centre, Ljubljana, Slovenia; 2 National Institute of Biology, Ljubljana, Slovenia; 3 Department of Knowledge Technologies, Jozef Stefan Institute, Ljubljana, Slovenia; 4 BioSistemika, raziskave in razvoj d.o.o., Ljubljana, Slovenia; 5 Educell Ltd., Trzin, Slovenia; 6 Department of Neurosurgery, Ljubljana University Medical Centre, University of Ljubljana, Ljubljana, Slovenia; 7 Faculty of Chemistry and Chemical Engineering, University of Ljubljana, Ljubljana, Slovenia; 8 Faculty of Pharmacy, University of Ljubljana, Ljubljana, Slovenia; 9 Celica d.o.o., Ljubljana, Slovenia; Dana-Farber Cancer Institute, UNITED STATES

## Abstract

**Background:**

Glioblastoma multiforme (GBM) is among the most aggressive cancers with a poor prognosis in spite of a plethora of established diagnostic and prognostic biomarkers and treatment modalities. Therefore, the current goal is the detection of novel biomarkers, possibly detectable in the blood of GBM patients that may enable an early diagnosis and are potential therapeutic targets, leading to more efficient interventions.

**Experimental Procedures:**

MicroRNA profiling of 734 human and human-associated viral miRNAs was performed on blood plasma samples from 16 healthy individuals and 16 patients with GBM, using the nCounter miRNA Expression Assay Kits.

**Results:**

We identified 19 miRNAs with significantly different plasma levels in GBM patients, compared to the healthy individuals group with the difference limited by a factor of 2. Additionally, 11 viral miRNAs were found differentially expressed in plasma of GBM patients and 24 miRNA levels significantly correlated with the patients’ survival. Moreover, the overlap between the group of candidate miRNAs for diagnostic biomarkers and the group of miRNAs associated with survival, consisted of ten miRNAs, showing both diagnostic and prognostic potential. Among them, hsa miR 592 and hsa miR 514a 3p have not been previously described in GBM and represent novel candidates for selective biomarkers. The possible signalling, induced by the revealed miRNAs is discussed, including those of viral origin, and in particular those related to the impaired immune response in the progression of GBM.

**Conclusion:**

The GBM burden is reflected in the alteration of the plasma miRNAs pattern, including viral miRNAs, representing the potential for future clinical application. Therefore proposed biomarker candidate miRNAs should be validated in a larger study of an independent cohort of patients.

## Introduction

Glioblastoma multiforme (GBM) is among the most aggressive and lethal types of brain cancer. The average life expectancy of GBM patients (GP) is 1.5 years despite current advanced treatment modalities and a plethora of established diagnostic biomarkers of the disease [1]. Thus for an earlier and more efficient GBM treatment, new diagnostic and prognostic markers are needed, that might also represent novel therapeutic targets [2]. Preferably, these should be detectable in easily accessible biological fluids using robust and minimally invasive methods.

In this respect, microRNAs (miRNAs), the small non-coding RNAs representing endogenous agents of RNA interference involved in the regulation of genes expression, emerge as suitable candidates [3,4]. Their overexpression, silencing or switching off was shown to affect GBM carcinogenesis via the deregulation of targeted oncogenes or tumor suppressor genes [5]. Circulating miRNAs found in human blood plasma may represent stable biomarkers as they are protected from degradation by being incorporated either into the lipid vesicles, i.e. microparticles and exosomes as well as in RNA-binding protein complexes [6,7]. Besides, plasma miRNA levels do not change substantially when kept at room temperature over a short period of time. Additionally, boiling and/or exposure to RNase A treatment, multiple freeze-thaw cycles and pH changes, also do not seem to affect the circulating miRNA structures [8–11]. This suggests that robust and stable circulating miRNAs could become potential clinical biomarkers [12].

To date, only limited analyses of the miRNA contents in peripheral blood have been performed. For example, in healthy individuals, gender-specific circulating miRNAs hsa—miR—548—3p, hsa—miR—1323, hsa—miR—940 and hsa—miR—1292 have been identified, whereas no differences in the miRNA content of microvesicles were revealed between different age groups [9,13,14]. In cancer tissue samples the pattern of miRNA expression differs from that of healthy individuals (HI), suggesting that miRNAs could play a critical role in the pathogenesis of this disease, as reviewed in [15]. Roth and coworkers were able to identify a specific miRNA signature in the blood cells of GBM patients (GP), namely an increased expression of hsa—miR—128 and hsa—miR—194 and a decreased expression of hsa—miR—342—3p and hsa—miR—628—3p [16]. In the microvesicles and plasma samples of GPs, decreased levels of hsa—miR—128 and hsa—miR—342—3p were determined, whereas those of hsa—miR—21 were found to be increased [17,18]. Until now, only a few miRNAs have been investigated in the plasma of GPs and all of those found to be differentially expressed have been previously reported present in GBM tissues [18,19]. To expand this knowledge, we performed a screening analysis of a large group of miRNAs in plasma samples of HIs and GPs. This approach could possibly lead to the discovery of novel plasma-specific miRNAs as candidates for early GBM diagnosis and as prognostic biomarkers.

Besides the host’s miRNAs, also some viral ones arising from latent infections are considered cancer development/progression promoters. Namely, viruses change the host miRNA expression profiles and make the infected cells more prone to oncogenic transformation. While certain viral miRNAs act in cis and regulate the virus genome expression in infected cells, possibly contributing to viral latency, those acting in trans, cause translational repression and/or a cleavage of the host cellular transcripts, normally involved in tumor suppression. Viruses also produce dsRNA-binding proteins that might act as suppressors of RNA silencing (VSRs) and interfere with the miRNA processor and effector complexes, thereby favouring tumor development. Some prediction studies even implicate that certain viral miRNAs directly regulate oncogenes. Some viral miRNAs may also facilitate tumor progression in uninfected tissues that are already prone to develop cancer. Such pro-oncogenic modulation is a typical characteristic of herpesviruses, for example EBV, HCMV, KSHV and HSV1. Altered host cellular miRNA repertoires found in cancerous tissues may influence their susceptibility to specific viruses. Thus tumor-specific changes in miRNA expression profiles could be responsible for the fact that specific viruses are often associated with a particular type of cancer regardless of their causal relationship [20]. Therefore, we aim to investigate whether some changes in viral miRNA plasma expression are representative for GBM.

For the identification of miRNA patterns in different biological samples, high throughput RNA expression profiling is generally carried out using microarrays, followed by real-time reverse transcriptase qPCR-based methods to validate the differentially expressed miRNAs [21]. In the last decade additional high-performance technologies have been developed, such as the nCounter technology [22]. Since the latter can be used to detect any type of nucleic acid in solution and be modified to assess other biological molecules as well, we chose it for the identification of putative new miRNA biomarkers screening in the plasma of GPs [22]. To our knowledge, this is the first study to address the profiling of plasma miRNA contents within the same individual over a defined time frame. Here we present a number of miRNAs, detected with novel screening analyses, as candidates for playing a role in GBM development. These also include viral miRNAs that have not been associated with GBM yet. Additionally, we present the use of extended SegMine methodology, linking changes in miRNA levels with consequently affected biological processes.

## Materials and Methods

### Plasma sampling

Glioblastoma (GBM) patients (GPs) were diagnosed and treated at the Department of Neurosurgery, Ljubljana University Medical Centre, Slovenia. The study was approved by the Republic of Slovenia National Medical Ethics Committee (Documents: 149/05/08 and 90/01/11). Upon obtaining signed informed consents, the blood samples were collected from 16 healthy individuals (HIs) recruited by the Blood Transfusion Centre of Slovenia in Ljubljana, and 16 GPs with a histopathological diagnosis of GBM (GPs). The inclusion criterion for HIs was their age (20–60 years) and their gender (half men, half women). The subjects with known acute and chronic diseases, pregnancy and those smoking and/or taking oral contraception or other drugs, were excluded from the study [23]. GPs had to fulfil the following inclusion criteria: age between 20 and 80 years and a confirmed diagnosis of a malignant glioma. The exclusion criteria for them were metastases and pregnancy. Each blood sample taken was screened for viral and bacterial infection markers of blood-transmittable diseases, i.e. HIV, hepatitis B, hepatitis C and syphilis, and in addition, the body mass index (BMI) was calculated for each individual enrolled.

Plasma samples of HIs were collected three times in the morning after overnight fasting, within a period of one month. The GP plasma samples were obtained prior to surgery, as well as 24 hours and one week after the surgery. Only three samples, collected 24 hours after the surgery, were included in the analysis. Another GP was included at the first occurrence of the disease and then once again at its remission, which was detected ten months after the first sampling. All GPs received pre- and postoperative corticosteroid therapy.

### MicroRNA extraction

Peripheral venous blood was drawn into the BD Vacutainer CPT Cell Preparation Tubes containing Sodium Citrate (Becton Dickinson Biosciences, USA). Upon centrifugation, the plasma aliquots were frozen and stored at -80°C until RNA extraction was performed using the mirVana Paris kit (Ambion, USA) according to a slightly modified manufacturer's instructions and with applying additional purification steps (see S1 File. MiRNA extraction protocol). The quality and quantity of the total RNA including small RNAs was assessed using a NanoDrop 8000 Spectrophotometer (Thermo Scientific, USA) and an Agilent 2100 Bioanalyzer, using the SmallRNA kit (Agilent Technologies, USA). The total RNA extracts were kept at -80°C until further processing.

### MicroRNA profiling

MiRNA expression profiling was performed using the nCounter miRNA Expression Assay Kits (NanoString Technologies, USA) at VIB Nucleomics (Belgium), allowing us to assay for a total of 734 human (654) and human-associated viral (80) miRNAs. The digital nCounter miRNA profiling technology is capable of accurately discriminating between miRNAs at a single-base resolution in a complex mixture. The system provides a direct digital readout of each miRNA needing only a small amount of the total RNA (100 ng), without requiring cDNA synthesis or enzymatic reactions. The method involves: mixing the total RNA with pairs of capture and reporter probes tailored to specifically recognize each miRNA present, hybridizing and washing away excess probes, immobilizing probe-bound miRNAs on a solid surface, and finally, scanning the different colour-coded bar tags on the reporter probes [24].

### Quantitative real-time PCR

The total RNA that was extracted as described (see MiRNA extraction), was used as a template for cDNA synthesis, qPCR reactions and data analyses, which were all performed according to the manufacturer's protocols. The total RNA (1–13 ng) was reverse transcribed using the TaqMan MicroRNA Reverse Transcription Kit (Applied Biosystems, USA) to quantify all the selected miRNAs, and with the High Capacity cDNA Reverse Transcription Kit (Applied Biosystems, USA) to assess the amounts of 18S rRNA. In these experiments, TaqMan 2x Universal PCR Master Mix, No AmpErase UNG and TaqMan MicroRNA Assays (Inventoried Assays, Applied Biosystems, USA) were used for the quantitative real-time PCR analyses of the following pre-selected miRNAs: ebv—miR—BART2—3p (Assay ID: 006174), ebv—miR—BART9—3p (Assay ID: 007435), hsa—miR—302c—3p (Assay ID: 000533), hsv1—miR—H1—5p (Assay ID: 464923_mat), hsa—miR—193a—3p (Assay ID: 002250), hsa—miR—503—5p (Assay ID: 001048) and hsa—miR—383—5p (Assay ID: 000573).

According to the different approaches that have been used in previously published experiments, three standard endogenous reference controls, i.e. the eukaryotic 18S rRNA TaqMan endogenous control (Assay ID: 4333760F), the small nuclear RNA RNU6B (Assay ID: 001093) and the hsa—miR—16 (Assay ID: 000391), were included in the analyses [25,26]. The RNU6B and hsa—miR—16 were analysed using the protocol and reagents for miRNAs, while in case of 18S rRNA, a 2x TaqMan Gene Expression Master Mix was used (Applied Biosystems, USA). Selected samples were processed in the set-up for quantitative PCR analysis using a LightCycler 480 Real-Time PCR System (Roche Applied Systems, USA), under the following conditions: enzyme activation at 95°C for 10 min, 40 cycles of denaturation (95°C, 15 sec) and the same number of steps of annealing and extension (60°C, 60 sec). The analysis of the resulting data was performed as described by Gruden et al. [23].

### Statistical analysis

The principal miRNA data analysis was performed using R software (http://www.r-project.org).

Since the sample volumes in the study were not completely balanced and the extraction efficiency varied from sample to sample, a proper normalization of results was eminent. Therefore a number of normalization protocols, including housekeeping stable miRNAs, the total miRNA content and the sum of positive controls, were tested. Based on the results obtained, normalization to the total miRNA content was chosen as the optimal data correction method. To reduce the problem with multiple testing in statistical models, the data were filtered for the nondetectable miRNAs (an expression value below the background in at least 80% of all samples) and also for hsa—miR—720 that is likely a fragment of a tRNA and was therefore removed from the miRBase [27]. Statistical analyses were performed on 284 miRNAs that met the upper criteria.

Hierarchical clustering analysis (HCA), a principal component analysis (PCA) and an analysis of variance were applied in order to get an overview of the sample variability, using the Multiexperiment Viewer software (MeV), where the criteria for data filtration were less strict [28]. Significantly altered miRNA plasma levels were statistically identified by the t—test or, in case of related samples, by the paired t—test.

In the survival analyses, the correlation between miRNAs detected in the plasma samples of GPs and their different survival times following the disease diagnosis was determined by the Kaplan-Meier statistics [29]. The optimal signal cut-off value that provided the most significant distinction between the sample groups was defined by stratifying the data according to six predetermined cut-off ratios: 0.25:0.75, 0.35:0.65, 0.45:0.55, 0.55:0.45, 0.65:0.35, and 0.75:0.25. The statistical significance of the differences (p—values) among these pre-defined group pairs was determined using the log-rank analysis. The relationships between the groups with the lowest p—values were represented by Kaplan-Meier graphs, following the application of corresponding functions within the R programme (R version 2.15.1, libraries KMsurv 0.1–5, knitr 1.2.10, patchDVI 1.9, survival 2.37–4).

### Development of the microRNA analysis workflow in SegMine

To allow the analysis of miRNA datasets and their targets we extended the SegMine methodology with special workflow components (widgets) which enable the analysis of miRNAs. Firstly, miRNA expression data in a comma separated format can be loaded by using the Data parser widget. Secondly, the Ranker widget ranks microRNAs according to their ability to distinguish between the two sets of samples (control and treatment sets) using the ReliefF algorithm [30]. Thirdly, the list of ranked microRNAs is translated into a ranked list of targets (genes). For this task the SegMine integrates three microRNA target databases: MiRTarBase with experimentally validated targets and two algorithmically predicted target databases, DIANA-microT-CDS and Targetscan [31–33]. The translation process is performed by voting where microRNAs contribute their ranks to their target genes. If available, target probabilities are used in the process, e.g. in the DIANA-microT-CDS the target probabilities are present for all the targets, while the Targetscan contains aggregate probabilities for only some of them. The translation step is followed by the unmodified SegMine workflow which performs a search for enriched gene sets with the SEGS algorithm, clustering of SEGS rules describing gene sets, and Biomine link discovery between the biological entities (e.g. selected genes, clusters of genes, rules, clusters of rules) [34]. This extension also enables adding the aggregated values from voting to the graph in order to easily identify important targets. Finally, the reverse inference to identify microRNAs which contributed to the observed target is also supported (if available, the target probabilities are taken into account).

We have taken the following steps using the extended SegMine to interpret the differences in miRNA levels between the HI older than 40 years (HIo) and GP groups: 1) the dataset was filtered for miRNAs with absolute logFC (log fold change) values lower than 0.2; 2) ranked miRNAs were translated into a ranked list of targeted genes based on the data from the miRTarBase—section Validated targets [31]; 3) SEGS rules with the corrected p-value ≤ 0.05 were taken into further analysis; 4) hierarchical clustering of gene ontology rules with Ward linkage criteria considering a cutoff line at 11.23 was used to generate six rule clusters that were subsequently analyzed individually, and 5) the resulting Biomine sub-graphs were comprehensively reviewed prior to focusing on the gene hubs (important nodes with a large number of edges).

## Results

### MicroRNA profiles in the plasma samples of healthy individuals (HIs)

**Fig 1:**
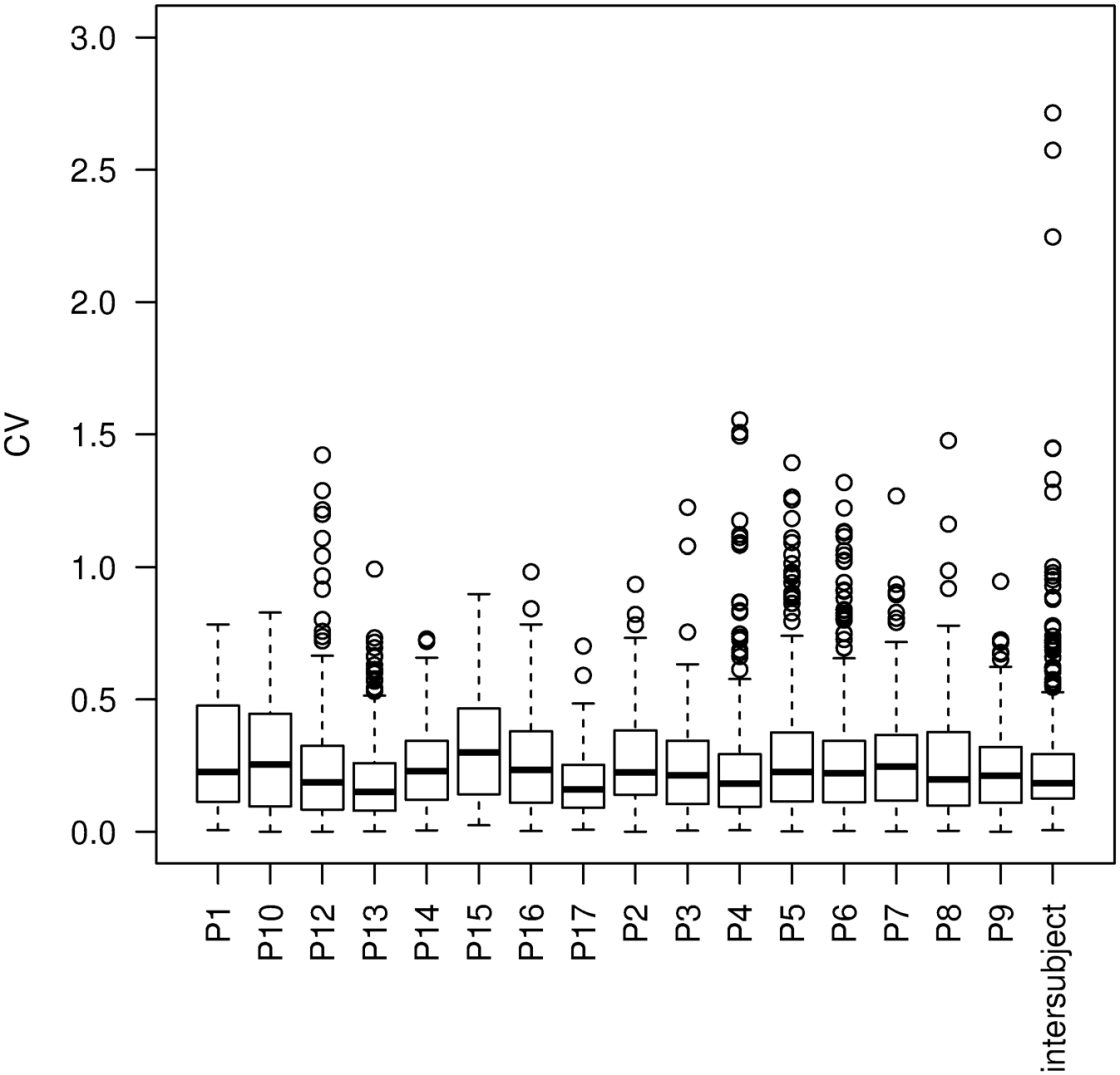
The comparison of intra- and inter-individual miRNA expression variability in the plasma samples of 16 HIs. Variabilities are represented as boxplots showing miRNA expression coefficients of variation (CVs) for each individual and a complete dataset.

MiRNA profiles in plasma samples were determined by using the nCounter technology. When conceiving the experimental design, special attention was paid to the sampling and sample processing in order to preserve miRNA stability and enable the standardization of the results. From the initial 734 miRNAs, 284 were included in the analyses following their normalization and omitting those below the detection threshold. If not specified otherwise, all the data were statistically evaluated by the t—test with a cut-off value of p = 0.01. In the group of HIs, the intra- and inter- individual variabilities of miRNAs were analyzed by using multivariate statistical methods (HCA, PCA). The coefficients of variation (CVs) represent the measure of physiological miRNA expression variability. Noteworthy, the assessed miRNA profiles showed greater intra—than inter-individual physiological variability, as presented in Fig 1.

The influence of BMI on individual miRNA profiles was tested in two groups of HIs, i.e. those with normal (≤24) and those having high (>24) BMI values. Only hsa—miR—323a—5p was significantly differently expressed, being elevated in a group of HIs with the higher BMI. Nevertheless, this miRNA was close to the limit of detection with only a few signals being positive in the group of HIs with the higher BMI (see S2 Table. The overview of the results). Therefore hsa—miR—323a—5p, despite emerging as differentially expressed, should only be potentially assumed to have a valid correlation with the elevated BMI.

Four miRNAs (hsa—miR—208a, hsa—miR—142—3p, hsa—miR—23a—3p and hsa—miR—1) were found to be age dependent (age < 40 versus age > 40 years) with the p—value < 0.05. All of them appeared decreased in the older subgroup of HIs. In a search of dissimilarities in miRNA expression between healthy men and healthy women, no differentially expressed miRNAs were found considering the p—value < 0.01. When the p—value < 0.05 was taken into account, ten miRNAs were found to be differentially expressed. Hsa—miR—1255a, hsa—miR—204, hsa—miR—651, hsa—miR—760 and kshv—miR—K12-4—5p were significantly increased, whereas hsa—miR—151—5p, hsa—miR—199a—5p, hsa—miR—361—5p, hsa—miR—423—3p and hsa—miR—423—5p, were significantly decreased within the male group of healthy individuals (HI).

Results of this part of research show trends that must be interpreted cautiously due to limited number of participants tested.

### Identification of differently expressed microRNAs in the plasma samples of GPs and HIs

In the search for novel biomarkers specific to GPs; plasma miRNA profiles of GPs and HIs were compared. Some miRNAs were detected exclusively in one of the two tested groups. Still, only those that were found to be under the detection threshold in less than 20% of samples within both groups were further analyzed. Differences in plasma miRNA levels within the two groups were statistically analyzed with the t—test or, in the case of related samples, (GP samples, obtained before and after surgery) by using the paired t—test, always with a cut-off value of p = 0.01 (adjusted p-value). To assimilate and neutralize the data with respect to age, the analyses were performed between all the GPs and only those HIs being older than 40 years (HIo). To avoid false positive results due to plasma contamination with erythrocytes, blood mononuclear cells and/or platelets, we systematically examined the results for the presence of miRNAs, characteristic for the hematopoietic system, based on the previous findings of several research groups [14,35,36]. These results are presented in Table 1. Together, 102 miRNAs, out of which 41 down-regulated and 61 up-regulated, were found with significantly different plasma levels in GPs, compared to the HIo group. These also included 11 viral miRNAs. After only miRNAs with at least a two-fold difference in levels detected in the HIo versus the GP group were considered among all the significantly different ones, 19 more potential candidates for miRNA biomarkers were revealed. The most strongly down-regulated miRNA in GPs was the hsa—miR—383 (logFC = −1.85), whereas the most strongly up-regulated one was the hsa—miR—603 (logFC = 2.68).

**Table 1:** MiRNAs with altered plasma levels in the GPs group vs. the HIo group.

ID	LogFC	GBM	Blood cells
hsa-miR-383-5p	-1.85	-	-
hsa-miR-660-5p	-1.82	-	**+**
hsa-miR-103a-3p	-1.62	-	**+**
hsa-miR-503-5p	-1.55	-	**+**
hsa-miR-320a	-1.33	-	**+**
hsa-miR-579-3p	-1.32	-	-
hsa-miR-302c-3p	-1.23	**+**	-
hsa-miR-492	-1.10	-	-
hsa-miR-508-3p	-1.05	-	-
hsa-miR-148b-3p	-1.03	-	**+**
hsa-miR-649	-1.00	-	-
hsa-miR-10b-5p	1.12	**+**	-
hsa-miR-487b-3p	1.13	-	**+**
hsv1-miR-H1-5p	1.16	NA	-
hsa-miR-613	1.31	-	-
hsa-miR-122-5p	1.49	-	-
hsa-miR-142-3p	1.54	**+**	**+**
hsa-miR-193a-3p	2.37	**+**	**+**
hsa-miR-603	2.68	-	-

The screening analysis was performed using the nCounter technology. MiRNAs with down—regulated plasma levels in the GP vs. the HIo group have the negative logFC values (fold change) and those up—regulated the positive logFC values (t—test, between subjects; just alpha; p<0.01), limited by the absolute logFC value of 1.0. The third column presents which miRNAs have already been correlated to GBM (glioblastoma) according to the miRTarBase [31]. Previous detection of explicit miRNA in the blood cells is marked in the fourth column [14,35,36]. Viral miRNAs are not included in the miRTarBase, therefore their correlation with GBM cannot be confirmed. A complete list of differentially expressed miRNAs is presented in S1 Table. A complete list of differentially expressed miRNAs. Legend: GP—patient with glioblastoma multiforme, HIo—healthy individual, older than 40 years, NA—not applicable;—and + label previously reported detection in GBM or in blood cells.

### Plasma microRNAs expression and GP survival

To determine whether the survival of GPs following GBM diagnosis associates with any particular plasma miRNA level, the log-rank analysis was performed. This enabled us to define the signal values for each miRNA that discriminated best between the short-term and long—term survivors. The analysis revealed 24 miRNAs significantly correlated with the survival of GPs. In a group of long-term survivors, 20 of them were found to be significantly up—regulated: hsa—miR—101—3p, hsa—miR—1260a, hsa—miR—145—5p, hsa—miR—185—5p, hsa—miR—18a—5p, hsa—miR—1913, hsa—miR—29c—3p, hsa—miR—302c—3p, hsa—miR—30a—5p, hsa—miR—30d—5p, hsa—miR—30e—5p, hsa—miR—483—5p, hsa—miR—484, hsa—miR—493—3p, hsa—miR—525—3p, hsa—miR—548d—3p, hsa—miR—548d—5p, hsa—miR—566, hsa—miR—592, hsa—miR—620, and four appeared to be significantly down-regulated: hsa—miR—124—3p, hsa—miR—155—5p, hsa—miR—514a—3p and hsa—miR—653—5p. The Kaplan-Meier graphs were generated for each of these 24 miRNAs (see S1 Fig. Kaplan-Meier graphs of 24 plasma miRNAs having an impact on GP survival (p-value < 0.05)). Red curves symbolize patients with miRNA signal intensities above the cut-off value, while the black curves represent those below it. Coloured areas mark the confidence intervals. It was noteworthy that no viral miRNA was found to be associated with the patients’ survival.

All MiRNAs with prognostic potential, according to the log-rank survival analysis, did not also hold diagnostic value, as they were not significantly differentially expressed in the GP and HIo plasma samples. However, ten miRNAs overlapped in this respect and were therefore considered to be both diagnostic and prognostic, which makes them particularly appealing. These were hsa—miR—302c—3p, hsa—miR—592, hsa—miR—484, hsa—miR—1260a, hsa—miR—493—3p, hsa—miR—514a—3p, hsa—miR—145—5p, hsa—miR—30a—5p, hsa—miR—124—3p and hsa—miR—483—5p.

### Analysis of the effects in microRNA changes at the level of cellular processes

The SegMine methodology was developed to enable the semantic analyses of gene expression data [37]. This was accomplished by integrating an advanced algorithm for gene set enrichment analysis (SEGS) and link the discovery within heterogeneous biological databases (Biomine) into a coherent data analysis workflow [38], [34]. We extended the SegMine methodology for the analysis of miRNA expression data at the level of cellular processes by linking miRNAs with their targets through available miRNA targets’ databases.

**Fig 2:**
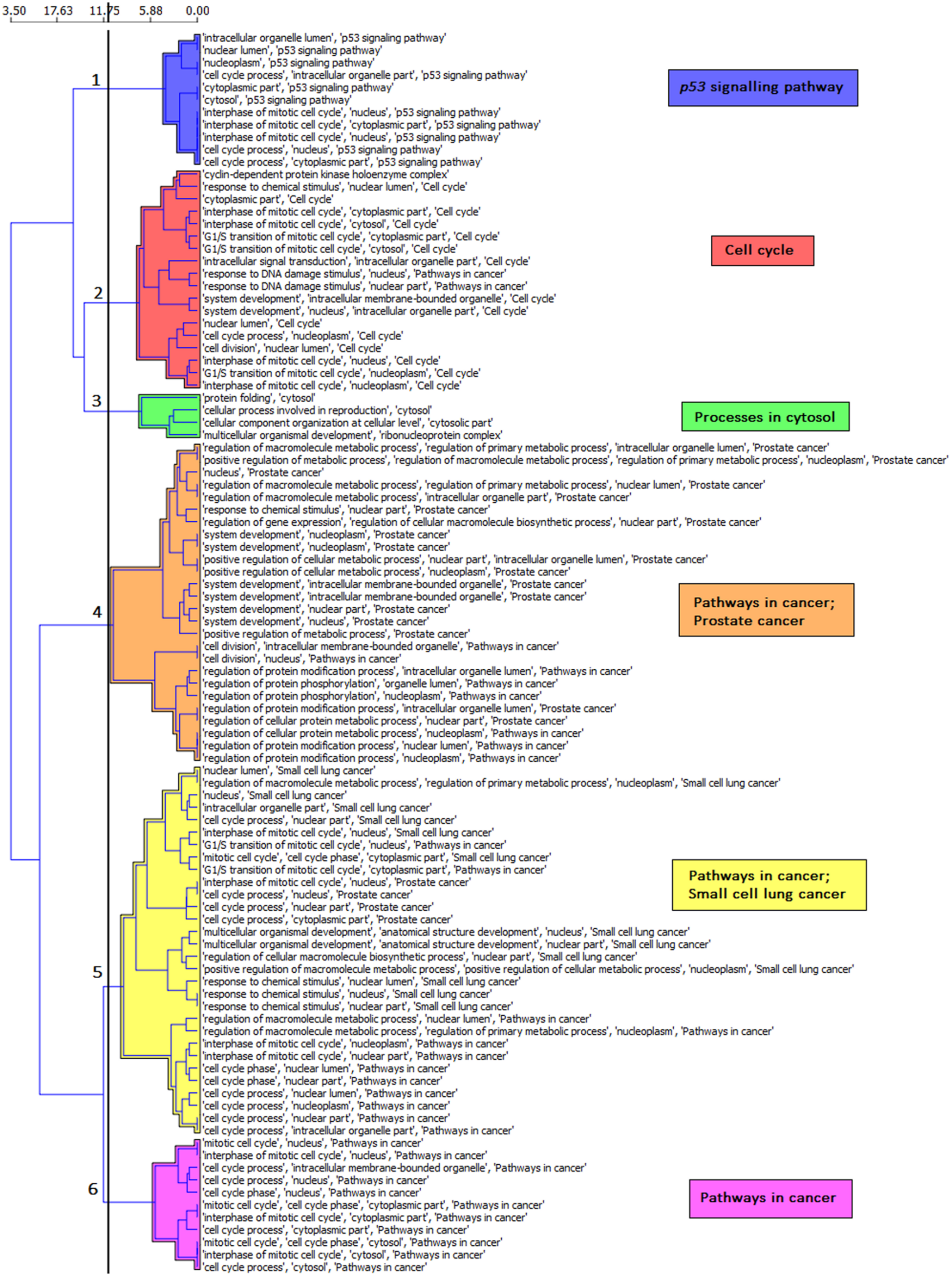
Hierarchical clusters of rules for the validated targets of miRNAs detected in the plasma samples of GPs. Hierarchical clustering of the top 100 statistically significant rules (p≤0.05) is presented. The SegMine rules were derived from genes, representing validated targets of the GBM-related plasma miRNAs. Euclidian distance and Ward’s linkage criteria were used to compute the hierarchy.

Six SEGS rule clusters describing the differences between the HIo and GP groups were generated using the miRNA analysis workflow within SegMine (Fig 2). As anticipated, the main miRNA clusters were linked with the gene ontology terms describing the cell proliferation processes or the ones involved in cancer signalling (Fig 2). Link discovery with Biomine revealed a generation of strong hubs, highly regulated by miRNAs that were found significantly differentially expressed between the HIo and GP groups. A thorough inspection of the Biomine sub-graphs revealed that the majority of gene hubs are cell cycle- or transcription-associated. Interestingly, one fraction of the nodes, having limited edges with very low weights, emerged to be linked to the main cluster. The fraction was found to cover the cytosolic processes, since the included genes code for numerous ribosomal proteins (see S2 Fig. The SegMine sub-graph showing a cluster of ribosomal proteins).

**Fig 3:**
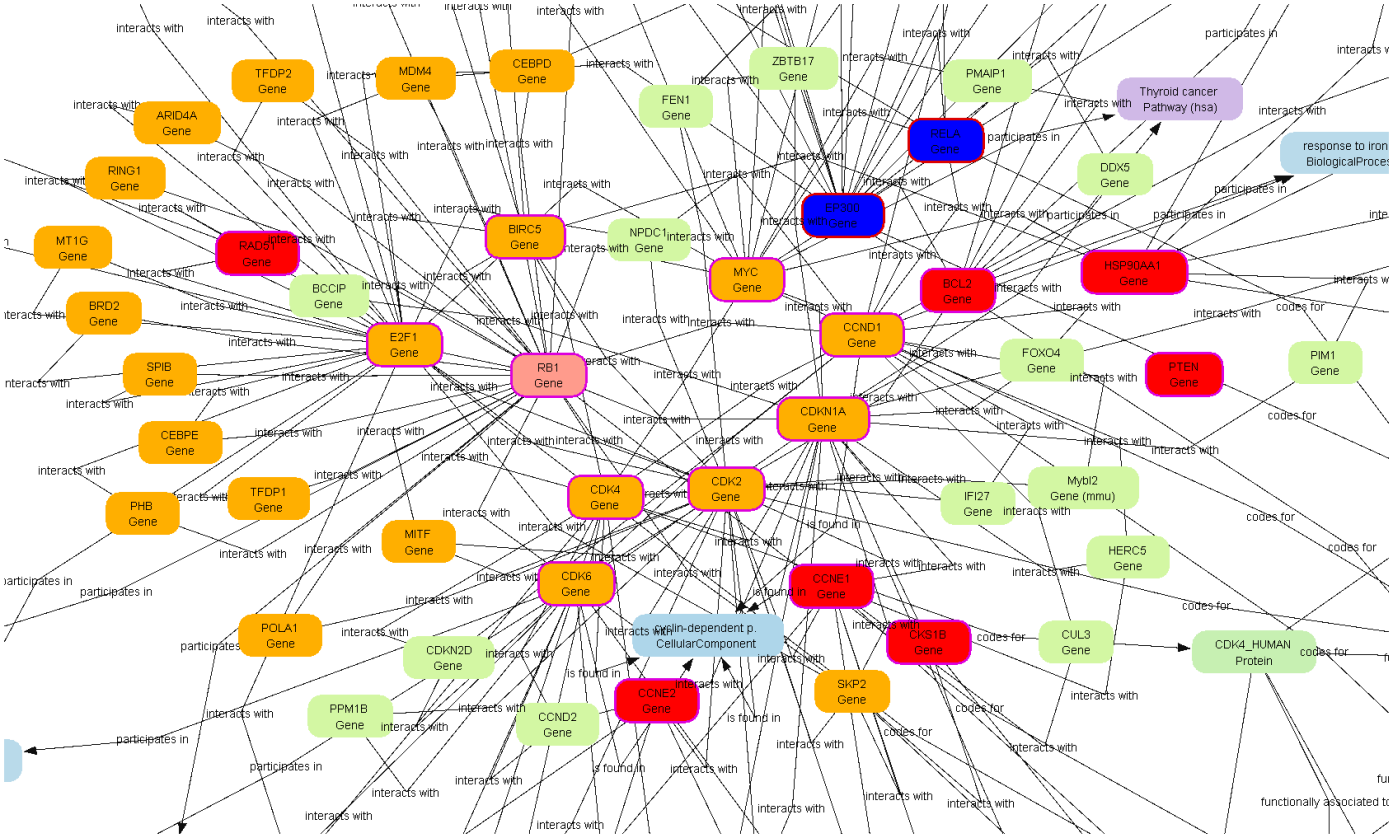
A Biomine sub-graph showing the Retinoblastoma (Rb) signalling pathway. Genes, involved in the RB signalling pathway are marked with an orange colour. The genes in the subgraph are covered by the rules from clusters 5 and 6 of the hierarchical clustering.

The intersection of the two Segmine clusters exposed proteins involved in the retinoblastoma (RB) signalling pathway; one of the most common and important mutated or deregulated pathways in GBM (Fig 3).

**Fig 4:**
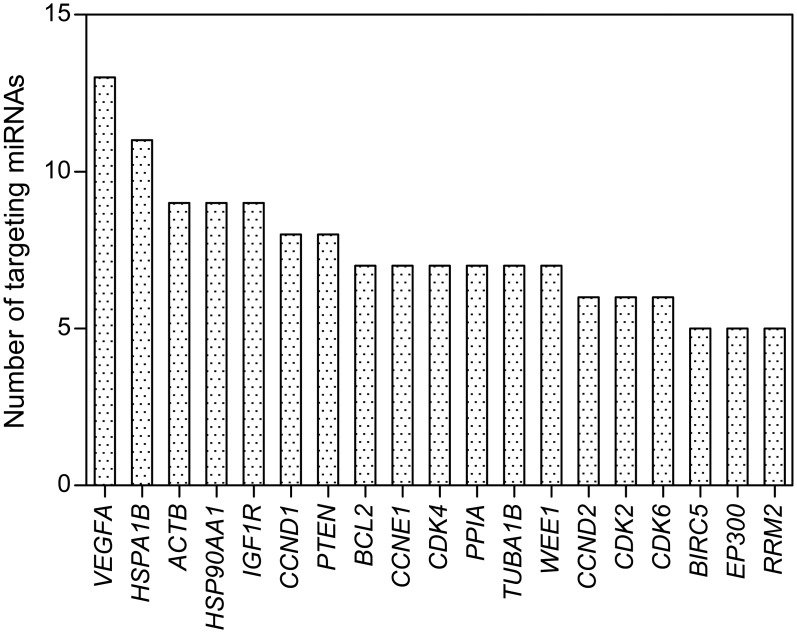
Genes most frequently targeted by miRNAs, correlated to the presence of GBM or patient survival. The presented genes are validated targets of miRNAs differentially expressed in the plasma samples of GPs and the members of the HIo subgroup and/or correlated to patient survival according to the results of analyses of this study, obtained by using the miRTarBase [32]. *VEGFA*—vascular endothelial growth factor A, *HSPA1B*—heat shock 70kDa protein 1B, *ACTB*—actin beta, *HSP90AA1*—heat shock protein 90kDa alpha (cytosolic), class A member 1, *IGF1R*—insulin-like growth factor 1 receptor, *CCND1*—cyclin D1, *PTEN*—phosphatase and tensin homolog, *BCL2*—B-cell CLL/lymphoma 2, *CCNE1*—cyclin E1, *CDK4*—cyclin-dependent kinase 4, *PPIA*—peptidylprolyl isomerase A (cyclophilin A), *TUBA1B*—tubulin, alpha 1b, *WEE1*—WEE1 G2 checkpoint kinase, *CCND2*—cyclin D2, *CDK2*—cyclin-dependent kinase 2, *CDK6*—cyclin-dependent kinase 6, *BIRC5*—baculoviral IAP repeat containing 5, *EP300*—E1A binding protein p300, *RRM2*—ribonucleotide reductase M2.

The genes most frequently targeted by miRNAs, differentially expressed in the plasma samples of GPs and HIos and/or correlated to GP survival were: vascular endothelial growth factor A (*VEGFA*), heat shock 70kDa protein 1B (*HSPA1B*), actin beta (*ACTB*), heat shock protein 90kDa alpha (cytosolic), class A member 1 (*HSP90AA1*), insulin-like growth factor 1 receptor (*IGF1R*), cyclin D1 *(CCND1*), as well as phosphatase and tensin homolog (*PTEN*) (Fig 4).

A reverse inference to identify miRNAs which have contributed to the observed target, supported by the SegMine methodology, provided us with a list of miRNAs, most frequently targeting genes included in the sub-graphs. Those targeting at least ten genes in the Biomine sub-graphs were: hsa—miR—320a, hsa—miR—484, hsa—miR—124—3p, hsa—miR—106b—5p, hsa—miR—103a—3p and hsa—miR—503—5p. All of them appeared correlated to the presence of GBM, with hsa—miR—484 and hsa—miR—124—3p being also associated with GP survival.

### Viral microRNAs found in the plasma samples of GPs and HIs

To find out whether viral miRNAs might have a role in GBM, a comparative analysis between GP and HIo plasma samples was performed for 80 human-associated viral miRNAs included in the nCounter miRNA expression assay. Eleven viral miRNAs were differentially expressed in the plasma samples of GPs, when compared to those of HIos. Six of them were Epstein Barr virus (EBV) miRNAs (ebv—miR—BART2-3p, ebv—miR—BART2-5p, ebv—miR—BART6-3p, ebv—miR—BART9, ebv—miR—BART15 and ebv—miR—BHRF1-3), 2 Herpes Simplex Virus (HSV) miRNAs (hsv1—miR-H1 and hsv1—miR-H4-3p), 1 Kaposi Sarkoma Human Virus (KSHV) miRNA (kshv—miR-K12-7) and two Human Cytomegalovirus (CMV) miRNAs (hcmv—miR-US5-2 and hcmv—miR-US33-3p).

**Fig 5:**
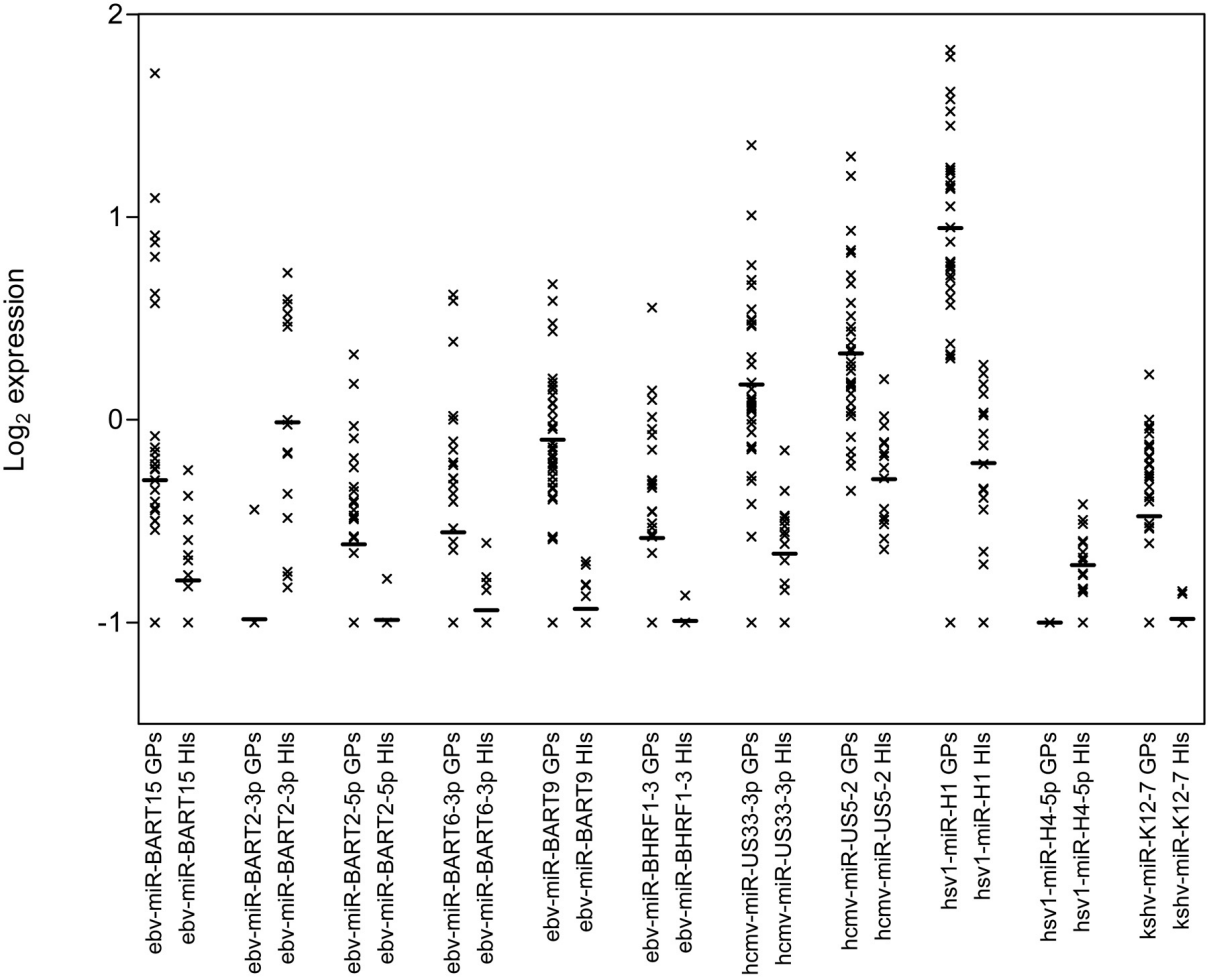
Log2 expression values of 11 viral miRNAs, differentially expressed in the GP and HIo plasma samples. The bars represent the mean values and x each analysed plasma sample.

Nine of them were up-regulated (ebv—miR—BART15, ebv—miR—BART2—5p, ebv—miR—BART6—3p, ebv—miR—BART9, ebv—miR—BHRF1-3, hcmv—miR—US5-2, hsv1—miR—H1 and kshv—miR—K12-7) and two (ebv—miR—BART2—3p and hsv1—miR—H4—5p) down-regulated in a group of GPs, compared to HIos (Fig 5).

These results suggest that viral miRNAs may play an important role in the tumorigenesis of GBM.

### Verification of nCounter technology results by real-time PCR (RT-qPCR)

RT-qPCR-based methods are mostly used for the validation of the results obtained with different microarrays and other high-throughput methods. For the confirmatory qPCR analysis, seven selected, differentially expressed miRNAs, ebv—miR—BART2—3p, ebv—miR—BART9—3p, hsa—miR—302c—3p, hsv1—miR—H1—5p, hsa—miR—193a—3p, hsa—miR—503—5p and hsa—miR—383—5p, were used. In addition, three standard endogenous reference controls, i.e. 18S rRNA, RNU6B and hsa—miR—16, were also included. No, or only a few signals were detected for ebv—miR—BART2—3p, ebv—miR—BART9—3p, hsa—miR—302c—3p, hsv1—miR—H1—5p, hsa—miR—193a—3p, hsa—miR—383—5p and RNU6B, while in the case of hsa—miR—503—5p, the results were not reproducible due to the high variability of the replicates. Reproducible results with the corresponding C_q_ values were obtained only for the endogenous reference controls 18S rRNA and hsa—miR—16. Although RT-qPCR methods for miRNA quantification are relatively inexpensive, commonly available and allow measurements of very small quantities of miRNAs, still the amounts of circulating miRNAs in the peripheral blood are often below their limit of detection [21]. Therefore, due to the low concentrations of circulating miRNAs found in the GP’s and HI’s plasma, RT—qPCR-based detection does not result in appropriate quantifications.

## Discussion

We performed a miRNA screening analysis in the plasma samples of GBM patients and healthy individuals using the nCounter technology and identified novel miRNA biomarker candidates for the diagnosis and prognosis of GPs. The majority (> 65%) of the differentially expressed miRNAs identified in this study had already been previously associated with various malignancies [39,40], many of them also with GBM [39,41]. The most down—regulated miRNA (hsa—miR—383) found in the investigated GP group may be involved in the promotion of glioma cell invasion by targeting the insulin-like growth factor 1 receptor gene (*IGF1R*) *via* AKT signalling that had been demonstrated previously [42]. In contrast, the hsa—miR—603 was found to be the most up-regulated miRNA in GPs when compared to the subgroup of older healthy individuals (HIo). It has not been previously linked to GBM, but its up-regulation improved the classification of gastrointestinal stromal tumors and is considered potentially oncogenic in bladder cancer [43,44]. Another miRNA with diagnostic potential is hsa—miR—10b, the increased expression of which had been repeatedly confirmed in GBM tissue [45–49], whereas Lang et al. also noticed this pattern in the cell lines originating from GBM stem cells [50]. Among miRNAs with the highest expression was also hsa—miR—193a—3p, previously correlated with a poor prognosis of GBM by Srinivasan et al. [50]. In the group of miRNAs with a decreased expression in the plasma of GPs, hsa—miR—302c—3p and hsa—miR—492 were discovered. The former was reported as down-regulated in GBM tumor samples, and the latter was decreased in GBM stem cell lines [48], [49]. Some inconsistencies regarding the expression between GBM tissue and plasma miRNAs were found, for example hsa—miR—181b and hsa—miR—181c were found down—regulated in the GBM tumor tissue, but are slightly up—regulated in the GP plasma, similar to the case of hsa—miR—124—3p [45–47]. These differences may be due to the poor secretion of some miRNAs from tissues/cells into the blood. Additionally, studies based on different blood fractions provide an overall picture of a patient’s condition, not exclusively related to the tumor burden. Besides, highly modified small RNA ends may cause a bias between the results obtained by hybridization-based methods versus the RT-qPCR [51]. Due to the very low miRNA concentrations in the plasma, we were faced with un-reproducible results of RT—qPCR, used for the validation of selected specific miRNAs, detected by the nCounter technology. Namely, we have decided to avoid amplification of cDNA that has been advised by other research groups due to possible errors and biases [42,43,52]. This study, as well as other authors, clearly demonstrated that the nCounter technology indeed provides superior gene expression quantification in human plasma, compared to the RT—qPCR [53].

Kaplan-Meier GP survival analyses revealed 24 plasma miRNAs with a prognostic potential in GBM. Twenty (20) upregulated and four downregulated miRNAs were linked to a longer survival of GPs. The overlap between the significantly upregulated miRNAs in the plasma of GPs compared to HIos and those with a prognostic value for GP survival, revealed a group of ten candidates, which might hold both, diagnostic and prognostic impact. Six of them (hsa—miR—302c—3p, hsa—miR—592, hsa—miR—484, hsa—miR—1260a, hsa—miR—493—3p and hsa—miR—514a—3p) were down-regulated and four (hsa—miR—145—5p, hsa—miR—30a—5p, hsa—miR—124—3p and hsa—miR—483—5p) were up-regulated in GPs. Among these, the association with GBM has already been confirmed for hsa—miR—302c—3p, hsa—miR—1260a, hsa—miR—145—5p, hsa—miR—30a—5p, hsa—miR—124—3p and hsa—miR—483—5p [47,53–57]. Moreover, the correlation of hsa—miR—484 and hsa—miR—493—3p with glioblastoma was questioned due to their presence within hematopoietic cells [14]. Two miRNAs, hsa—miR—592 and hsa—miR—514a—3p have not been previously associatied with the GBM, though the first one has been correlated to other cancers, for example medulloblastoma [43,58].

To expose the most significant, we propose hsa—miR—592 and hsa—miR—514a—3p as potential novel GBM biomarkers with both, diagnostic and prognostic clinical potential. Validated target genes of hsa—miR—592 are *DICER* (dicer 1, ribonuclease type III), an endonuclease with a role in the biogenesis of the active small RNA component and *CCND1* that interacts with the Rb tumor suppressor protein [59]. Mutations, amplification and overexpression of this gene, which alters the cell cycle progression, are frequently observed in a variety of tumors, including a subtype of GBM and may well contribute to its tumorigenesis [39,60]. Significantly altered expressions of hsa—miR—592 have been observed among tumors with deficient, as well as proficient mismatch repair in colon cancer [61,62]. In contrast to these two, no correlation with cancer and no gene targets have been reported for the hsa—miR—514a—3p variant so far [39].

For the purpose of this study we decided to upgrade SegMine, the new bioinformatic methodology, to allow for the visualization of meaningful correlations among miRNAs and their target genes. In this way, genes strongly associated with differentially expressed miRNAs in the plasma of GPs were identified and found to be part of a transcriptional regulation network (*E2F1-* E2F transcription factor 1, *MYC*—v-myc avian myelocytomatosis viral oncogene homolog, *RELA*—v-rel avian reticuloendotheliosis viral oncogene homolog A), cell cycle progression (*CDK2*, *CDK4*, *CDK6*, *CCND1*, *CCND2*, *CCNE1*, *CDKN1A -* cyclin-dependent kinase inhibitor 1A, *PTEN*, *RB1*), apoptosis (*BCL2*, *BIRC5*) and stress regulation (*HSPA1B*, *HSP90AA1*) proteins. There, both identified miRNA candidates for diagnostic and prognostic biomarkers, the hsa—miR—124—3p and hsa—miR—484, again appeared among the miRNAs, whose gene targets were most numerously represented.

The group of miRNAs with the most striking differences detected in the plasma of GPs were viral miRNAs. Viral miRNAs allow for viral persistence in the host by inhibiting the cell-mediated immunity, apoptosis and cell-cycle, thereby providing conditions favouring latency [63]. These mechanisms may link the viral infection to tumorigenesis, where similar processes are deregulated. Yet, the question remains, whether viral miRNAs may also affect uninfected host cells. This was demonstrated for the functionally active ebv—miR—BHRF1—3 found inside exosomes released from EBV infected cells, consequently causing a down-regulation of its target gene *CXCL-11* (Chemokine (C-X-C) Motif Ligand 11) also in the non-infected ones [64]. This study revealed 11 differentially expressed viral miRNAs in the plasma of GPs, compared to those of HIs. Six (6) of them belong to the Epstein Barr virus (EBV) miRNAs, and the rest are of Herpes Simplex Virus (HSV), Kaposi Sarkoma Human Virus (KSHV) and Human Cytomegalovirus (CMV) origin. Except for ebv—miR—BART2-3p and hsv1—miR-H4—3p, all the others were up-regulated in the plasma samples of GPs.

A control over the latency to lytic transition may enable viruses to evade the anti-viral host immune responses and may act in a similar manner in tumors, helping to evade immune surveillance during tumor progression. EBV miRNAs were demonstrated to target and control the cellular and viral synthesis of proteins involved in the transition from the latency to lytic state. The ebv—miR—BART2 represses the viral DNA polymerase BALF5, thereby maintaining its latency, while its reduced levels diminish the BALF5 transcript cleavage and allow for EBV lytic replication [65]. In GP plasma samples hsv1—miR-H1 was also up-, whereas hsv1—miR-H4-5p was down-regulated. Both, hsv1—miR-H1, which is exclusively expressed during productive infection, and hsv1—miR-H4, which is known to down-regulate the viral pathogenicity factor ICP34.5, inhibit the innate immune response to HSV-1 and promote its replication within the neurons [66]. Likewise, the decreased levels of ebv—miR—BART2-3p miRNA along with the increased levels of HSV—1 and CMV miRNAs found in the plasma samples of GPs could affect the immune reponse in GBM patients [67,68].

Furthermore, to evade the immune response, viral miRNAs may also be interfering with the immune cell mediated cytotoxicity. Ebv—miR—BART2-5p, found increased in plasma samples of GPs, targets the 3'UTR of MICB, a stress-induced non-classical major histocompatibility class I molecule, thereby reducing the cytotoxic death of the infected cells, mediated by MICB binding to the NKG2D receptors expressed on the natural killer (NK) and CD8^+^ T cells. As tumor cells also express MICB, the ebv—miR—BART2-5p, may be in this way involved in GBM progression [65]. Additionally, the ebv—miR—BHRF1-3 miRNA, also found increased in GP plasma, is known to directly down-regulate the host chemokine CXCL-11. Since this is produced by B lymphocytes following IFN—γ stimulation, and upon binding to its CXCR3 receptors activates the NK and T cells, the induction of these important immune cells may also be impaired in GPs [63,65]. Likewise, KSHV miRNAs play similar roles in modulating the expression of the same genes. In the plasma samples of GPs, significantly higher levels of kshv—miR-K12-7 were detected, as in those of HIs. Kshv—miR—K12-7, like ebv—miR—BART2—5p, is known to repress MICB expression. Additionally, it down-regulates the expression of the liver inhibitory protein (LIP), which causes cell death, stimulates autophagy and activates the transcription and secretion of IL-6 and IL-10 in monocytes and macrophages [69]. These cytokines play an active role in the pathogenesis of KSHV-associated malignancies by promoting tumor growth, angiogenesis and suppressing T—cell activation [70]. So putatively, the kshv—miR—K12-7 may also function similarly in GBM progression.

With respect to cell death, EBV miRNAs inhibit apoptosis in cell lines during their transfection with the BART (BamHI A rightward transcripts) miRNA coding region. Multiple predicted sites for binding cluster I and cluster II BART miRNAs exist within the Bcl-2 interfacing mediator of the cell death gene (BIM). Its encoded BIM tumor suppressor protein synthesis is down-regulated by the expression of both miRNA clusters [71,72]. We found ebv—miR—BART6 and ebv—miR—BART15 (Cluster I), as well as ebv—miR—BART9 (Cluster II) up-regulated in the plasma of GPs. BIM is also known to be targeted by cellular miRNAs in nasopharyngeal and gastric carcinomas [71]. Additional predicted target genes for ebv-miR-BART6-3p, ebv—miR—BART9 and ebv—miR—BART15 are CASP10, RAD1/RB1, and CASP3, respectively [73]. Moreover, the up-regulated ebv—miR—BHRF1-3, found in the GP plasma samples, was shown to match BID (BH3 interacting domain death agonist) and BHRF (Bam HI fragment H rightward open reading frame, a homolog of mammalian BCL2) transcripts, which, when silenced, block the extrinsic and mitochondrial apoptotic pathways, the lower cell’s responsiveness to external signals and increase its resistance to apoptosis [70]. Besides BID, EBV miRNAs may also negatively target the pro-apoptotic protein Cyclin G2 (CCNG2), which is down-regulated in different tumors, where these two key components of the p53 pathway may be regulating cell cycle arrest, senescence and apoptosis [73]. The abundance of these EBV miRNAs in the plasma of GPs could thus be supporting the increased apoptotic resistance of GBM.

In this study we have also tested whether age, gender, physical activity and BMI correlate with the detected miRNA expression patterns in the plasma samples of GPs and HIs. We identified four age-related miRNAs (p<0.05). All of them were present at lower levels in the older group of HIs (HIo). Two of them, hsa—miR—208a and hsa—miR—1 have a common validated target gene *CASP3* that plays a central role in the execution phase of cell death and may therefore be related to age [74]. Hsa—miR—142—3p, hsa—miR—23a—3p and hsa—miR—1 also all target the *CCNDI* oncogene [39]. The down-regulation of miRNAs targeting this gene can thus lead to enhanced cell cycle progression and telomere shortening that after a certain time/age may result in tumorigenic transformation-prone cell evolution.

## Conclusion

The presented analysis of human plasma-derived miRNAs in glioblastoma patients *vs* normal healthy individuals provides a broader insight into the potential of miRNAs to be used as specific biomarkers for GBM diagnosis and prognosis using the novel nCounter technology, being more sensitive than the classical methods. We have found 19 differentially expressed miRNAs with a minimal 2-fold difference in the levels detected in the tested groups of which not all have a prognostic value and also 24 miRNAs which fulfil this criterium but were not significantly altered in the plasma of GPs compared to normal HIs. Besides, a subgroup of ten miRNA candidates was defined that hold both, diagnostic and prognostic value for GBM patients. The most interesting result of this study was a differential expression of 11 viral miRNAs, which are known to modulate the cell cycle, and repress apoptosis and immune responses. Their influence is in concert with the signals generated by specific tumor-protecting microenvironment and altogether immune and neuroendocrine system responses in cancer patients. However, a broader clinical validation of the proposed biomarker candidate miRNAs requires further studies in an independent cohort of patients.

## Supporting Information

S1 Fig Kaplan-Meier graphs of 24 plasma miRNAs having an impact on GP survival (p-value < 0.05). (DOC)

S2 Fig The SegMine sub-graph showing a cluster of ribosomal proteins. (DOC)

S1 File MiRNA extraction protocol. (DOC)

S1 Table A complete list of differentially expressed miRNAs. (DOC)

S2 Table The overview of the results. (XLSX)

S3 Table Raw data. (XLSX)
