# Supplementary material for: Analysis of Glioblastoma Patients' Plasma Revealed the Presence of MicroRNAs with a Prognostic Impact on Survival and Those of Viral Origin
Source: PLoS One. 2015 May 7;10(5):e0125791. doi: 10.1371/journal.pone.0125791 (PMC4423889; doi:10.1371/journal.pone.0125791)
Supplement: S1 Fig — (DOC) [file pone.0125791.s001.doc]

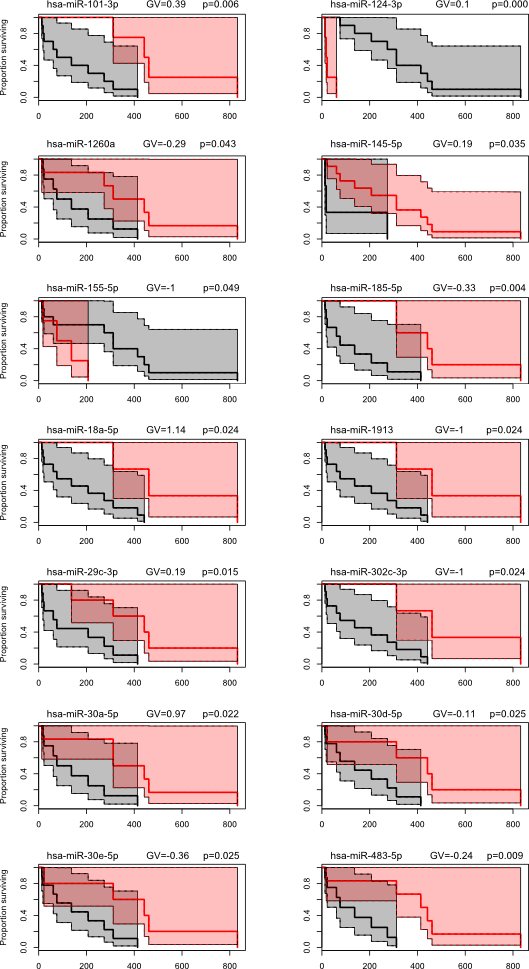


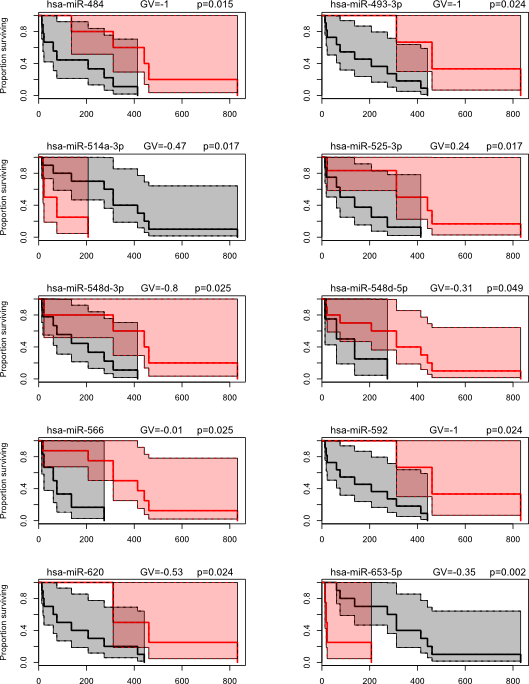


S1 Fig. Kaplan-Meier graphs of 24 plasma miRNAs having impact on GP survival (p‑value < 0.05). Red curves symbolize patients with signal intensities above the cut-off value, while the black curves represent patients with signal intensities below it. Colored areas mark the confidence intervals. On the X-axes the survival time of patients (in days) following diagnosis, and on the Y-axes the cumulative proportions (%) of survivors are displayed, respectively.
