# Supplementary material for: Analysis of Glioblastoma Patients' Plasma Revealed the Presence of MicroRNAs with a Prognostic Impact on Survival and Those of Viral Origin
Source: PLoS One. 2015 May 7;10(5):e0125791. doi: 10.1371/journal.pone.0125791 (PMC4423889; doi:10.1371/journal.pone.0125791)
Supplement: S2 Fig — (DOC) [file pone.0125791.s002.doc]

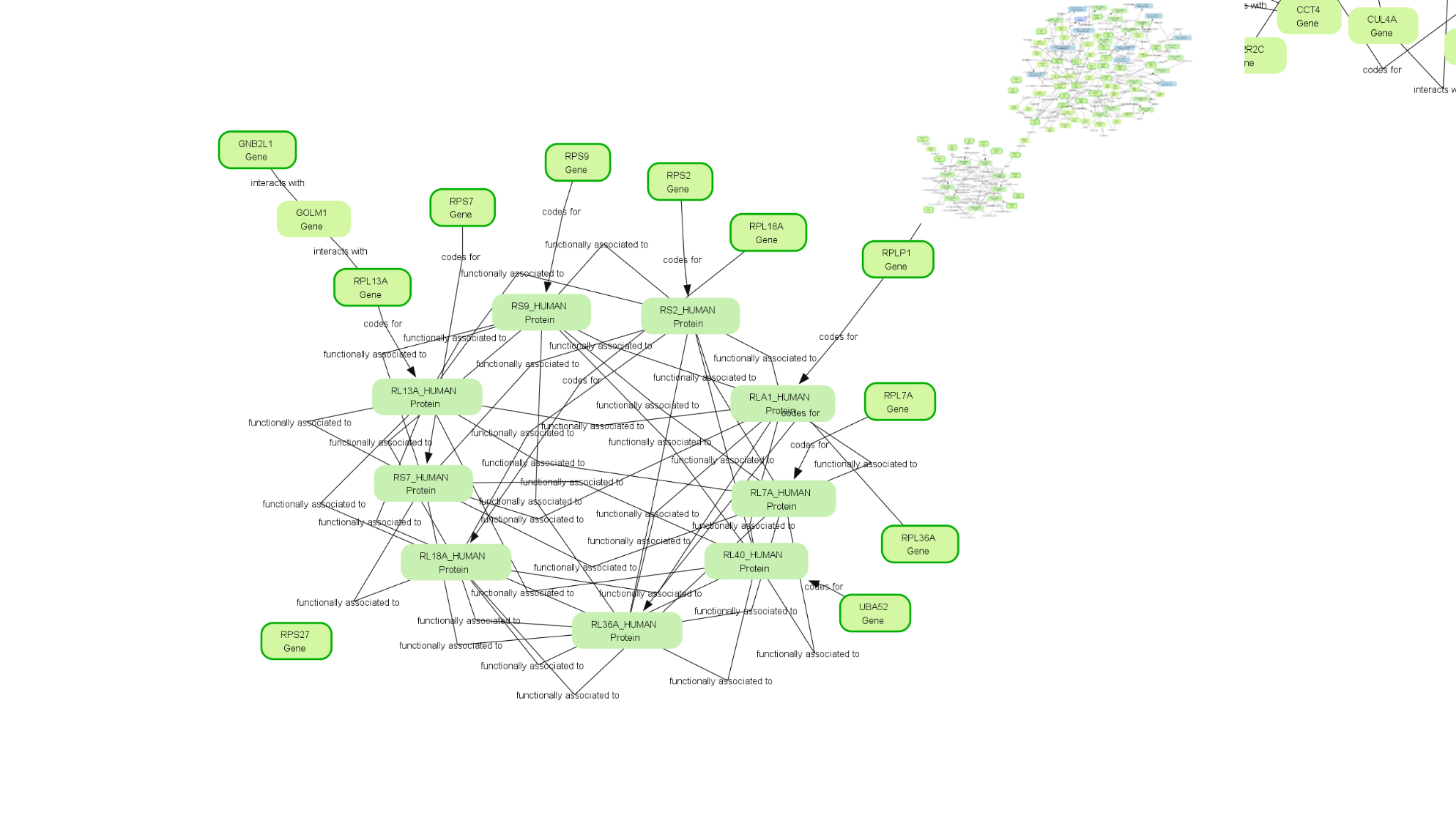


S2 Fig. A Biomine sub-graph showing a cluster of ribosomal proteins. A part of the sub-graph pertaining to the cluster of cytosolic processes, with a strict separation from the main cluster, in which an exclusive linkages of genes, coding for ribosomal proteins is clearly shown.
