# Supplementary material for: Analysis of Glioblastoma Patients' Plasma Revealed the Presence of MicroRNAs with a Prognostic Impact on Survival and Those of Viral Origin
Source: PLoS One. 2015 May 7;10(5):e0125791. doi: 10.1371/journal.pone.0125791 (PMC4423889; doi:10.1371/journal.pone.0125791)
Supplement: S1 File — (DOC) [file pone.0125791.s003.doc]

Ambions' mirVana PARIS kit manual with a few modifications

Organic extraction:

- Thaw the plasma on ice.
- Mix with an equal volume of 2x denaturing buffer, incubate on ice for 5 minutes.
- Add a volume of acid-phenol: ChCl3 equal to the sample and denaturing buffer together, vortex and allow to sit for another 2 minutes at room temperature.
- Centrifuge at 16000 x g for 10 minutes. When solid interphase is not formed, this step is repeated.

Isolation of small RNAs:

- Draw off the top aqueous phase, determine the volume and add 1.25 volumes of 100% EtOH.
- Mix well and load the sample onto a filter 700 μl at a time, running each volume through for 30 seconds at 10,000 x g.
- Wash the filter 5 times:

a) 700 μl Wash solution 1

b) 500 μl Wash solution 2/3

c) 500 μl Wash solution 2/3

d) 500 μl 80% ethanol

e) 500 μl 80% ethanol.

- After removing the final flow through, spin the column again at 10000 x g for 2 minutes.
- Transfer the column to a new tube and elute in two steps with 80 μl of 95°C water.

Additional step for purification and concentration of samples:

- Add 1/10 volume 3M sodium acetate pH 5.2.
- Add 1μl of carrier (1mg/ml linear acrilamide).
- Add 2.5 volumes ice-cold 95% EtOH. Leave on ice for 30 minutes.
- Spin top speed in refrigerated microfuge for 30 minutes at 4°C.
- Discard supernatant carefully, add 200μl ice-cold 75% EtOH. Do not disturb pellet; spin at 4°C for 15 minutes.
- Dry pellet and resuspend in small volume of RNAse-free water.
