# Supplementary material for: Analysis of Glioblastoma Patients' Plasma Revealed the Presence of MicroRNAs with a Prognostic Impact on Survival and Those of Viral Origin
Source: PLoS One. 2015 May 7;10(5):e0125791. doi: 10.1371/journal.pone.0125791 (PMC4423889; doi:10.1371/journal.pone.0125791)
Supplement: S1 Table — (DOC) [file pone.0125791.s004.doc]

S1 Table. MiRNAs with altered plasma levels in the GPs group vs. the HIo group. The screening analysis was performed with nCounter method. MiRNAs with down‑regulated plasma levels in the GPs group vs. the HIo group have negative log FC (fold change) and up‑regulated have positive log FC (t‑test, between subjects; just alpha; p<0.01), limited by absolute log FC value of 0.2. The third and the seventh column presents which miRNAs have already been correlated to GBM (glioblastoma) according to miRTarBase [35]. Previous detection of explicit miRNA in blood cells is marked in fourth and eighth column [15], [45], [46]. Viral miRNAs are not included in miRTarBase, therefore the correlation with GBM cannot be confirmed. (GP – patient with glioblastoma multiforme, HIo – healthy individual, older than 40 years, NA – not applicable; - and + label detection in GBM [35] or detection in blood cells [15], [45], [46]).

| ID | Log FC | GBM | Blood cells |
| --- | --- | --- | --- |
| hsa-miR-383-5p | -1.85 | - | - |
| hsa-miR-660-5p | -1.82 | - | **+** |
| hsa-miR-103a-3p | -1.62 | - | **+** |
| hsa-miR-503-5p | -1.55 | - | **+** |
| hsa-miR-320a | -1.33 | - | **+** |
| hsa-miR-579-3p | -1.32 | - | - |
| hsa-miR-302c-3p | -1.23 | **+** | - |
| hsa-miR-492 | -1.10 | - | - |
| hsa-miR-508-3p | -1.05 | - | - |
| hsa-miR-148b-3p | -1.03 | - | **+** |
| hsa-miR-649 | -1.00 | - | - |
| hsa-miR-592 | -0.99 | - | - |
| ebv-miR-BART2-3p | -0.93 | NA | - |
| hsa-miR-378a-3p | -0.91 | - | **+** |
| hsa-miR-24-3p | -0.90 | - | **+** |
| hsa-miR-150-5p | -0.86 | - | **+** |
| hsa-miR-106b-5p | -0.85 | - | **+** |
| hsa-miR-376a-3p | -0.85 | **+** | **+** |
| hsa-miR-600 | -0.82 | - | - |
| hsa-miR-30b-5p | -0.82 | - | **+** |
| hsa-miR-484 | -0.72 | - | **+** |
| hsa-miR-519e-3p | -0.72 | - | - |
| hsa-miR-425-5p | -0.69 | **+** | **+** |
| hsa-miR-496 | -0.69 | - | - |
| hsa-miR-147a | -0.66 | - | - |
| hsa-miR-328-3p | -0.60 | **+** | **+** |
| hsa-miR-1260a | -0.57 | **+** | - |
| hsa-miR-370-3p | -0.56 | - | **+** |
| hsa-miR-760 | -0.54 | - | - |
| hsa-miR-493-3p | -0.48 | - | **+** |
| hsa-miR-548i | -0.47 | - | - |
| hsa-miR-491-3p | -0.46 | **+** | **+** |
| hsa-miR-576-3p | -0.43 | - | - |
| hsa-miR-1255a | -0.43 | - | **+** |
| hsa-miR-641 | -0.41 | - | **+** |
| hsa-miR-514a-3p | -0.39 | - | - |
| hsa-miR-519d-3p | -0.37 | - | - |
| hsa-miR-222-3p | -0.34 | **+** | **+** |
| hsa-miR-515-3p | -0.33 | - | - |
| hsv1-miR-H4-5p | -0.30 | NA | - |
| hsa-miR-488-3p | -0.22 | - | - |
| ebv-miR-BHRF1-3 | 0.26 | NA | - |
| hsa-miR-1305 | 0.30 | **+** | - |
| hsa-miR-154-5p | 0.32 | - | **+** |
| hsa-miR-1243 | 0.34 | - | - |
| hsa-miR-181b-5p | 0.36 | **+** | **+** |
| hsa-miR-616-3p | 0.37 | - | - |
| hsa-miR-145-5p | 0.38 | **+** | **+** |
| hsa-miR-326 | 0.38 | **+** | **+** |
| hsa-miR-195-5p | 0.39 | **+** | **+** |
| hsa-miR-342-5p | 0.41 | **+** | **+** |
| ebv-miR-BART6-3p | 0.41 | NA | - |
| hsa-miR-598-3p | 0.43 | - | **+** |
| ebv-miR-BART2-5p | 0.43 | NA | - |
| hsa-miR-640 | 0.43 | - | - |
| hsa-miR-19b-3p | 0.43 | **+** | **+** |
| hsa-miR-380-3p | 0.45 | - | - |
| hsa-miR-1203 | 0.46 | - | - |
| hsa-miR-30a-5p | 0.47 | **+** | **+** |
| kshv-miR-K12-7-3p | 0.47 | NA | - |
| hsa-miR-323a-5p | 0.47 | - | - |
| hsa-miR-26b-5p | 0.48 | - | **+** |
| hsa-miR-140-5p | 0.49 | - | **+** |
| hsa-miR-200b-3p | 0.50 | **+** | - |
| hsa-miR-626 | 0.51 | - | **+** |
| hsa-miR-409-5p | 0.52 | - | **+** |
| hsa-miR-555 | 0.53 | - | - |
| hsa-miR-570-3p | 0.54 | - | - |
| hsa-miR-124-3p | 0.54 | **+** | - |
| hsa-miR-29a-3p | 0.55 | **+** | **+** |
| hcmv-miR-US5-2-3p | 0.55 | NA | - |
| hsa-miR-1266-5p | 0.56 | - | - |
| hsa-miR-181c-5p | 0.58 | **+** | **+** |
| hsa-miR-483-5p | 0.59 | - | - |
| hsa-miR-548b-3p | 0.60 | - | - |
| hsa-miR-1244 | 0.62 | - | - |
| hsa-miR-652-3p | 0.63 | - | **+** |
| hsa-miR-330-5p | 0.67 | - | **+** |
| hsa-miR-584-5p | 0.67 | - | **+** |
| hsa-miR-877-5p | 0.67 | - | **+** |
| hsa-miR-1279 | 0.72 | - | - |
| hcmv-miR-US33-3p | 0.72 | NA | - |
| hsa-miR-517c-3p | 0.74 | - | - |
| hsa-miR-15a-5p | 0.76 | **+** | **+** |
| hsa-miR-601 | 0.78 | - | - |
| hsa-miR-885-3p | 0.78 | **+** | - |
| hsa-miR-1297 | 0.79 | - | - |
| hsa-miR-1307-3p | 0.80 | - | **+** |
| hsa-miR-361-3p | 0.81 | - | **+** |
| hsa-miR-759 | 0.82 | - | - |
| ebv-miR-BART15 | 0.82 | NA | - |
| hsa-miR-10a-5p | 0.83 | **+** | - |
| hsa-miR-1226-3p | 0.94 | - | - |
| ebv-miR-BART9-3p | 0.95 | NA | - |
| hsa-miR-10b-5p | 1.12 | **+** | - |
| hsa-miR-487b-3p | 1.13 | - | **+** |
| hsv1-miR-H1-5p | 1.16 | NA | - |
| hsa-miR-613 | 1.31 | - | - |
| hsa-miR-122-5p | 1.49 | - | - |
| hsa-miR-142-3p | 1.54 | **+** | **+** |
| hsa-miR-193a-3p | 2.37 | **+** | **+** |
| hsa-miR-603 | 2.68 | - | - |
